# Comprehensive Analysis of miRNAs and Target mRNAs between Immature and Mature Testis Tissue in Chinese Red Steppes Cattle

**DOI:** 10.3390/ani11113024

**Published:** 2021-10-21

**Authors:** Xibi Fang, Lihong Qin, Haibin Yu, Ping Jiang, Lixin Xia, Zhen Gao, Runjun Yang, Yumin Zhao, Xianzhong Yu, Zhihui Zhao

**Affiliations:** 1 College of Animal Science, Jilin University, Changchun 130062, China; fangxibi@jlu.edu.cn (X.F.); xialx18@mails.jlu.edu.cn (L.X.); yrj@jlu.edu.cn (R.Y.); 2 Branch of Animal Husbandry, Jilin Academy of Agricultural Sciences, Changchun 130033, China; qlhqlh2007@163.com (L.Q.); yumin_zh@163.com (Y.Z.); 3 College of Coastal Agricultural College, Guangdong Ocean University, Zhanjiang 524088, China; yuhb@gdou.edu.cn (H.Y.); jiangp@gdou.edu.cn (P.J.); gzhen1203@126.com (Z.G.); 4 Department of Biological Sciences, Clemson University, Clemson, SC 29634, USA

**Keywords:** cattle, male reproduction, microRNA, functional gene

## Abstract

**Simple Summary:**

MicroRNAs are small molecules that can regulate the relative abundance of their target genes by binding to the 3′ untranslated region of the target genes at the post-transcriptional level to affect various biological processes, such as biosynthesis, fat metabolism and proliferation, apoptosis, and cell differentiation. Fertility is one of the most important economic traits in livestock production. Bulls require the continuous production of high-quality spermatozoa in abundance. The quality of semen is an exceptionally important factor affecting the fertilization rate of the dairy cow and is also associated with the increasing conception rate in the process of artificial insemination. Therefore, accurately predicting fertility potential for a semen sample from donor bull for artificial insemination is crucial for consistently high reproductive efficiency. The present study performed a genome-wide sequencing analysis of microRNAs and mRNAs between immature and mature testes of Chinese Red Steppes. These results provide novel candidate microRNAs and functional genes related to bull reproduction traits and the networks between microRNAs and target genes, which will provide a useful genetic mechanism and epigenetic information for marker-assisted selection of bulls with excellent sperm quality in the future.

**Abstract:**

This study aims to screen potential regulators and regulate fecundity networks between microRNAs (miRNAs) and target genes. The bovine testes of immature and mature Chinese Red Steppes were performed by genome-wide analysis of mRNAs and miRNAs. Compared with testicular tissues of newborns, 6051 upregulated genes and 7104 downregulated genes in adult cattle were identified as differentially expressed genes (DEGs). The DEGs were significantly enriched in 808 GO terms (*p* < 0.05) including male gonad development, male genitalia development, spermatogenesis, and sperm motility. Moreover, DEGs were also significantly enriched in 105 KEGG pathways (*p* < 0.05), including cGMP-PKG signaling pathway and calcium signaling pathway. To explore the expression of miRNA-regulated gene expression, 896 differentially expressed target genes negatively regulated with the expression levels of 31 differentially expressed miRNAs (DERs) were predicted and analyzed, and a network-integrated analysis was constructed. Furthermore, real-time PCR was performed to verify the expression levels of DEGs and DERs. Our results identified novel candidate DEGs and DERs correlated with male reproduction and intricate regulating networks between miRNAs and genes, which will be valuable for future genetic and epigenetic studies of sperm development and maturity, as well as providing valuable insights into the molecular mechanisms of male fertility and spermatogenesis in cattle.

## 1. Introduction

As the site for spermatogenesis, the mammalian testis is crucial in male reproduction [1]. Mammalian spermatogenesis begins at puberty and results in the formation of spermatozoa, a unique population of haploid cells via three continuous stages, including constant self-renewal of spermatogonia, meiotic division of spermatocytes, and post-meiotic differentiation of haploid spermatids [2,3]. These three unique events are strictly regulated by stage-specifically expressed genes at both transcriptional and post-transcriptional levels [4,5]. Compared to other organ-specific transcriptomes with about 200 signature genes, testis exhibits a more complex transcriptional profile with more than 15,000 genes expressed [6,7,8], and the previous RNA-seq data of humans and mice showed that the brain, and especially the testis, express more protein-coding genes than others organs [7]. It has also been suggested that non-coding RNAs, such as microRNAs (miRNAs), long non-coding RNAs (lncRNAs), circular RNAs (circRNAs), and Piwi-interacting RNAs (piRNAs), function as important regulators of gene expression at the post-transcriptional level in spermatogenesis [9,10,11,12]. Furthermore, somatic cells such as Sertoli cells and Leydig cells are important in testis formation and form a nurturing and regulatory environment for spermatogenesis in mice and rats [13,14]. However, the majority of coding and non-coding transcripts involved in each stage or cell type, as well as their functions are yet to be annotated.

Fertility is one of the essential traits of reproduction in bulls, and accurate prediction of fertility potential for a semen sample from donor bull for artificial insemination is crucial for consistently high reproductive efficiency [15]. Over the years, assays have been developed for semen quality prediction based on various spermatozoal attributes, such as motility and morphology, chromatin structure, capacitation and/or the acrosome, zona-free hamster egg penetration reaction, in vitro fertilization of homologous oocytes, membrane integrity, and various semen fluid proteins [16,17,18,19,20,21,22,23]. However, the diagnostic test for fertility based on a single attribute is not accurate and consistent which could potentially lead to devastating economic loss.

With the advancement of RNA-seq technology, mRNA and non-coding RNA in the testes have been profiled in various animal species [24,25,26,27,28,29,30], which have provided insights into the molecular mechanisms governing testis development and spermatogenesis. Furthermore, various mRNAs and non-coding RNAs have been identified as associated with the quality of sperm fertility [31]. Therefore, studying the differential expression of mRNA and miRNA between mature and immature bull testes might identify new directions for fertility biomarker discovery as well as new targets for infertility treatments.

Chinese Red Steppes are a Chinese indigenous cattle breed from northeast China, bred as dual-purpose cattle. They have unique features such as disease resistance and better meat quality than other local Chinese yellow cattle. Due to the rich and extensive genetic resources, Chinese Red Steppes have received much attention from cattle genetics and breeding researchers in China.

In this study, we performed genome-wide profiling of mRNA and miRNA of Chinese Red Steppes cattle by next-generation sequencing technology in immature and mature testicular tissues. Differentially expressed genes (DEGs) were screened between two groups, and the Gene Ontology (GO) terms and Kyoto Encyclopedia of Genes and Genomes (KEGG) pathways enrichment were analyzed to classify the function of DEGs. Meanwhile, to understand the function of DEGs, GO terms tree, KEGG pathways act-networks, and regulated networks were generated, respectively. These results provided key miRNAs and functional genes in the process of bovine male reproduction which will provide useful information for marker-assisted selection of bulls with excellent sperm quality in the future.

## 2. Materials and Methods

### 2.1. Ethics Statement

Animal care and experiments were performed according to the guidelines established by the care and use of laboratory animals of the Jilin University Animal Care and Use Committee (Permit number: SY201901007).

### 2.2. Animal and Samples

A total of 6 bovine testicular lobules tissues of 3 each for 1-day old castrated calves and 24-months-old slaughtered adult bulls were collected from the Agricultural Science Academy of Jilin Province cattle farm (Gongzhuling, China). The testicular tissues of castrated calves were defined as immature testicular tissues group (I) whereas those of the slaughtered adult bulls were defined as mature testicular tissues group (M).

### 2.3. RNA Extraction and Quality Analysis

Total RNA was extracted from the collected testicular lobules tissues using trizol reagent (Invitrogen, Waltham, MA, USA) following the manufacturer’s instructions. Total RNA was treated with DNase I (NEB, Beijing, China). The RNA concentration and integrity quality were detected using an Agilent 2100 Bioanalyzer (Agilent Technologies, Palo Alto, CA, USA) and the RIN or RNA integrity number was used to determine the RNA integrity quality of extraction.

### 2.4. Library Preparation of mRNA and miRNA and Quantification

Six libraries of RNA-seq were constructed and grouped to I and M by the source of testicular lobules tissues for subsequent analysis. mRNA was enriched with oligo (dT) beads and fractionated for cDNA synthesis. The cDNA was then amplified to prepare the mRNA library. Library concentration was quantified by qPCR and a Qubit^®^ 2.0 Fluorometer (Life Technologies, Carlsbad, CA, USA), and the insert size was checked on an Agilent Bioanalyzer 2100 system (Agilent Technologies, Palo Alto, Santa Clara, CA, USA). The cDNA libraries were sequenced using the Illumina HiSeq2000 platform by the Beijing Genomics Institute (BGI, Shenzhen, China).

Two small RNA libraries representing each group (from the pooling of testis tissues in 3 newborns and 3 adult cattle) were constructed for Solexa sequencing according to the Illumina^®^ TruSeq™ Small RNA Sample manufacturer’s instructions. The Agilent 2100 system was used to determine the library size and purity. The small RNA libraries were finally sequenced using Solexa sequencing by BGI.

### 2.5. Analysis of Differentially Expressed Genes

The raw sequencing data were also evaluated by Fast-QC (http://www.bioinformatics.babraham.ac.uk/projects/fastqc/ (accessed on 8 March 2018)), which included the quality distribution of nucleotide to help us understand the natural characteristics of data before subsequent evaluation. The clean reads were aligned to the bovine genome (Btau_5.0.1) using the Hisat2 software. According to our data, the EB-Seq algorithm was applied to filter differentially expressed genes for the 6 samples in immature from mature testes groups. After significance analysis and false discovery rate (FDR) analysis [32,33], we selected the DEGs according to the Log2^FC^ > 1 or <−1, FDR < 0.05.

### 2.6. GO and KEGG Enrichment Analysis of DEGs

DEGs were implemented by the GOseq R package [34]. GO was used to determine and compare the functions of the DEGs as biological process, molecular function, and cellular component, with corrected *p* values of less than 0.05 considered significantly enriched. Association of the genes with pathways was computed with the KEGG (http://www.genome.jp/kegg (accessed on 8 March 2018)) [35,36,37]. A pathway with a corrected *p*-value < 0.05 was considered as significantly enriched. Next, the pathway network was generated using the KEGG database based on the relationships between different genes [38].

### 2.7. Analysis of miRNA Profiling and Prediction of Novel miRNA

The original small RNA reads were processed with Fast-QC (http://www.bioinformatics.babraham.ac.uk/projects/fastqc/ (accessed on 8 March 2018)) to remove adaptor sequences and low-quality sequences. The small RNA reads were aligned with Sanger miRBase (version 21.0, https://mirbase.org/ (accessed on 8 March 2018)) [39] and the data were compared to the cattle genome (Btau_5.0.1) by BWA software (http://bio-bwa.sourceforge.net/ (accessed on 8 March 2018)). The novel miRNA was predicted using the aligned reads and unmapped reads were extracted and mapped to the miRBase databases of human and mouse genome (version 21.0, https://mirbase.org/ (accessed on 8 March 2018)) [39]. Furthermore, like the algorithm of DEGs, the EB-seq algorithm was also employed to filter the differentially expressed miRNAs (DERs) based on FC and FDR thresholds [33]. MiRNAs were considered as differentially expressed according to the Log2^FC^ > 1 or <−1, FDR < 0.05.

### 2.8. miRNA Target Gene Prediction and Annotation Analyses

For association analysis, both RNAhybrid (https://bibiserv.cebitec.uni-bielefeld.de/rnahybrid (accessed on 8 March 2018)) and miRanda (http://www.microrna.org/ (accessed on 8 March 2018)) programs were used to discover the potential miRNA target [40,41]. Furthermore, the DEGs and DERs were integrated to determine whether the expression levels of each miRNA and its mRNA targets were negatively correlated (Li et al., 2011). The miRNA target which had no negative correlation analysis with the DERs and DEGs were filtered out. To build the miRNA-Gene-Network between DERs and DEGs, the adjacency matrix of miRNA and genes A = (ai,j) was made by the attributed relationships among genes and miRNA, and ai,j represents the relation weigh of gene i and miRNA j.

### 2.9. Real-Time PCR of DEGs and DERs

Real-time PCR was utilized to measure the expression levels of DEGs and DERs of 6 samples, respectively. Three biological replicates were included in each group, and 3 technical replicates were performed for each sample. The reverse transcription primers and primers of DEGs for quantitative analysis were designed using Primer 6.0 (Premier Biosoft International, Canada), and the stem-loop primers of DERs were designed according to the method reported [42]. U6 small nuclear RNA was used as an internal reference of miRNAs, and *β-actin* was used as a reference to detect relative expression of mRNAs. All the sequence information is shown in Table S1. According to the manufacturer’s instructions, 1 µg total RNA was used to synthesize cDNA with the PrimeScript™ RT reagent Kit with gDNA Eraser (Takara Bio Inc., Dalian, China). Real-time PCR was performed using PCRmax Eco 48 (PCRmax, Staffordshire, UK). The real-time PCR system was as follows: 5 μL FastStart Universal SYBR Green Master, 1 μL cDNA, 0.2 μL primer-F (10 μM), 0.2 μL primer-R (10 μM) and 3.6 μL RNase-free water. Reactions were incubated at 95 °C for 10 min, followed by 40 cycles of 95 °C for 10 s and 60 °C for 30 s. Relative expression levels were calculated using the 2^−ΔΔCt^.

## 3. Results

### 3.1. Differential Expression of mRNAs in Chinese Red Steppes between Immature and Mature Testicular Tissues

**Figure 1:**
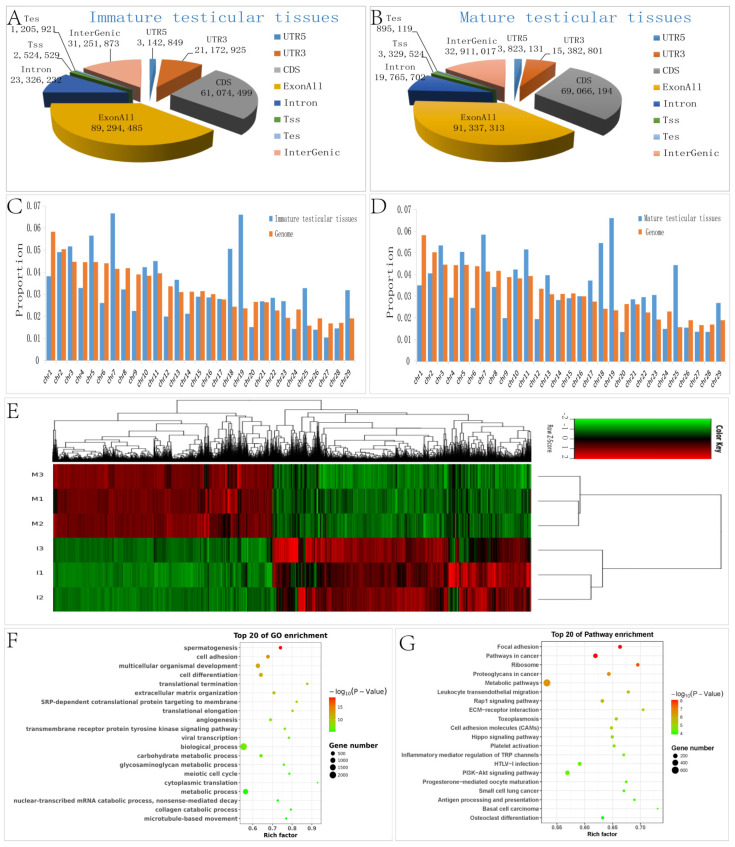
Analysis of RNA-seq data and differentially expressed genes. (**A**,**B**) Functional genomic elements distribution of mRNA reads in immature testicular tissue and mature testicular tissue, each part of the pie chart representing the number of average reads enriched into each functional element; (**C**,**D**) The average distribution coverage at different chromosomes of the genome about mapped reads in immature testicular tissue and mature testicular tissue. The blue bar charts show the proportion of average distribution coverage, and the gray bar charts show the proportion of different chromosomes lengths in the genome; (**E**) Heat map of differentially expressed genes, and the color bar represent normalized FPKM values, green represents down-regulation of gene mRNA expression levels, and red represents up-regulation; (**F**) GO enrichment of differentially expressed genes; (**G**) KEGG enrichment of differentially expressed genes. **Note:** The ordinate represents the enriched GO terms and pathways, and the abscissa represents the rich factor of corresponding pathways; the size of the spots represents the number of differentially expressed genes enriched in each GO term and pathway, while the color of the spot represents the corrected *p* value of each pathway. The rich factors indicate the ratio of the number of DEGs mapped to a certain GO terms or pathway to the total number of genes mapped. Greater rich factor means greater enrichment.

A total of 321,537,748 reads were obtained from 6 samples. After filtering out low-quality data, 317,756,254 clean reads were mapped to *Bos Taurus* (Btau_5.0.1). High-quality maps of the two bovine groups were obtained and the unique mapping rates ranged from 85.7% to 87%. The details of the sequencing data quality of mRNA are shown in Table S1. Gene structure analysis was performed for each sample (Figure 1A,B) and the distribution of the reads on the chromosomes is shown in Figure 1C,D. The sequence data is submitted to the GEO (Gene Expression Omnibus) with the accession number GSE137464.

Comparing the results of immature with mature testes, a large number of DEGs were identified of which 6051 DEGs were up-regulated and 7104 were down-regulated including tripartite motif-containing 42 (*TRIM42*), sperm acrosome associated 4 (*SPACA4*), family with sequence similarity 187 member B (*FAM187B*), and phosphoribosyl pyrophosphate synthetase 1-like 1 (*PRPS1L1*). The 15 most differentially expressed up- and 15 most differentially expressed down-regulated genes between the immature and mature testicular tissue are listed in Table 1 and the heat map of all DEGs is shown in Figure 1E.

### 3.2. GO and KEGG Pathway Analyses of DEGs

**Figure 2:**
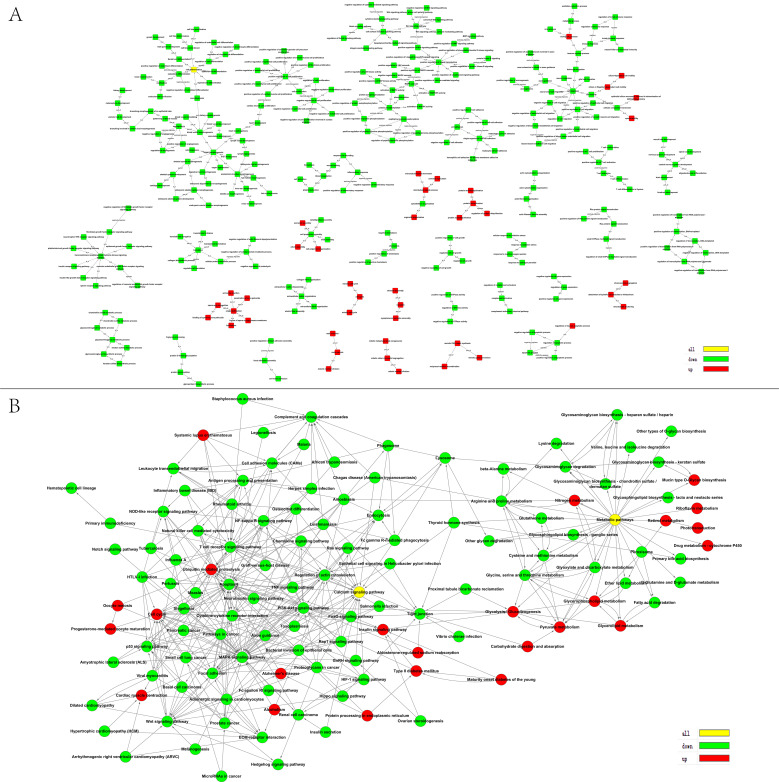
Diagram GO tree and KEGG act network of upregulated genes and downregulated genes enriched. (**A**) GO tree; (**B**) KEGG act network. **Note:** Red represents the GO term or KEGG pathway corresponding to the up-regulated gene, green represents the GO term or KEGG pathway corresponding to the down-regulated gene, and yellow indicates the GO term or KEGG pathway corresponding to both the up-regulated and down-regulated genes.

GO analysis of functional enrichments of DEGs found that 9056, 1226, and 3576 GO terms were enriched in biological processes, molecular functions, and cellular components, respectively. 525 biological processes, 124 molecular functions, and 159 cellular components were significantly enriched (*p* < 0.05). The most significantly enriched functional terms included spermatogenesis (GO:0007283), cell adhesion (GO:0007155), and multicellular organismal development (GO:0007275). The most significantly enriched biological processes include calcium ion binding (GO:0005509), protein binding (GO:0005515), and molecular function (GO:0003674). Extracellular matrix (GO:0031012), proteinaceous extracellular matrix (GO:0005578), and extracellular vesicular exosome (GO:0070062) are among the most significantly enriched cellular components. The top 20 GO terms are listed in Figure 1F by ascending order of corrected *p* value and the regulating network of significant GO terms (*p* < 0.01) are shown in the GO tree (Figure 2A).

KEGG analysis of functional enrichments of 3331 DEGs found that 282 pathways were enriched, with 105 pathways significantly enriched (*p* < 0.05). These included calcium signaling pathway (PATH:04020), metabolic pathways (PATH:01100), cell adhesion molecules (PATH:04514), and PI3K–Akt signaling pathway (PATH:04151). The top 20 pathways are listed in Figure 1G by the ascending order of *p*-value. Furthermore, the regulating network of significant pathways is shown in Figure 2B. Networks constructed by most KEGG pathways of down-regulated genes showed both up- and down-regulated genes enriched in calcium signaling pathway and metabolic pathways, and were similar to the GO tree in that most up-regulated genes were enriched in ubiquitin-mediated proteolysis.

### 3.3. Analysis of miRNAs Expression Patterns in Testicular Tissues

**Figure 3:**
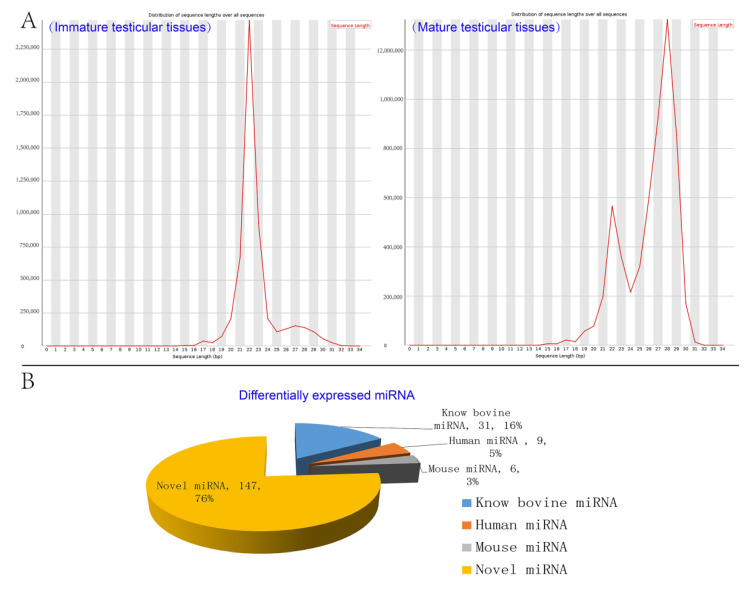
Analysis of miRNAs data by Solexa sequencing. (**A**) Length distribution of the clean reads; (**B**) Number and percentage of identified miRNAs.

Solexa sequencing yielded a total of 11,091,870 clean reads for the two groups. The length distribution of clean reads indicated that the immature group was mainly around 22nt to 24nt in size while the mature group was mostly 22nt and 28nt in size (Figure 3A). 97.2% and 98.5% were mapped to the bovine genome in immature and mature libraries, respectively. There were 77.7% clean reads of immature group data mapped to the known miRNA database, whereas this was only 17.1% clean reads for the mature group. The details of the Solexa sequencing of miRNA data are shown in Table S2.

Between immature and mature testis tissues of Chinese Red Steppes, 31 differentially expressed bovine miRNAs, nine human miRNAs, six mouse miRNAs, and 147 unknown miRNA were screened (Figure 3B). Among the bovine miRNAs, 11 miRNAs were up-regulated and 20 were down-regulated, including bta-miR-34b, bta-miR-34c, bta-miR-485, and bta-miR-449a. All 31 differentially expressed bovine miRNAs are listed in Table 2.

### 3.4. Prediction and Construction of Target Network between DERs and DEGs

**Figure 4:**
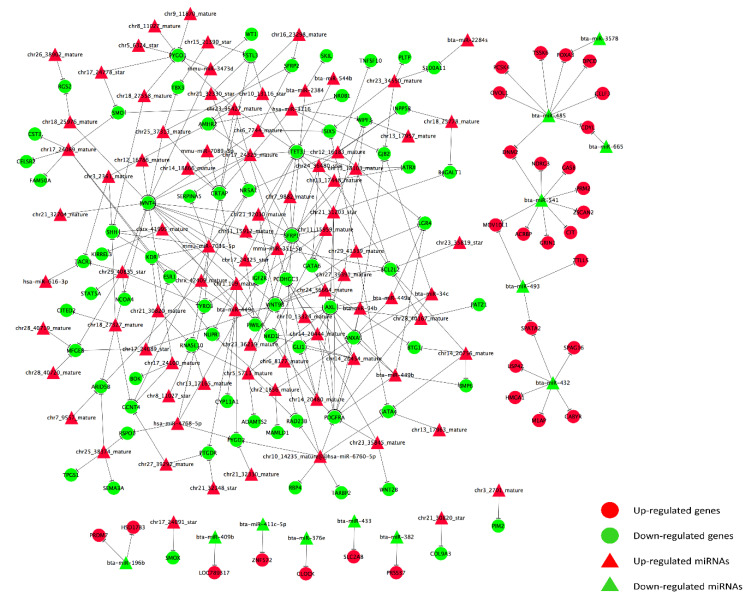
Network of miRNAs and target genes prediction and annotation analyses. Green triangles: Downregulated miRNAs; Red triangles: Upregulated miRNAs; Green circles: Downregulated genes; Red circles: Upregulated genes.

**Figure 5:**
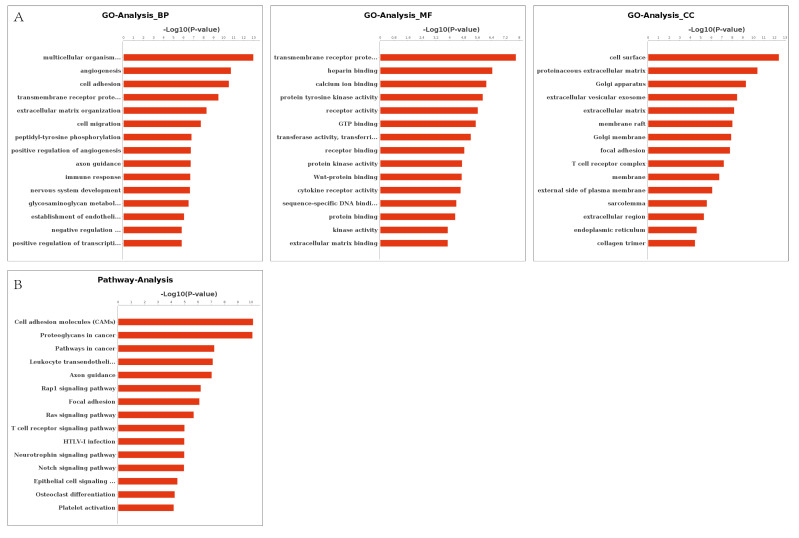
Significantly enriched GO terms and KEGG pathways. (**A**) Significantly enriched GO terms of target genes of differentially expressed miRNA; (**B**) Significantly enriched KEGG pathways of target genes of differentially expressed miRNA.

DERs and their target DEGs were identified by integrative analysis and the result showed that 896 DEGs were target genes of 31 differentially expressed bovine miRNAs. Their targeting networks are shown in Figure 4. Overall, target genes of identified DERs were located in 10,023 GO terms, of which 1092 terms were significantly enriched (*p* < 0.05). These included regulation of sperm motility (GO:1901317) sperm motility (GO:0030317), spermatogenesis (GO:0007283), and spermatid development (GO:0007286), all of which play vital roles in male fecundity. KEGG analysis showed that 1352 target genes of DEGs were distributed among 277 pathways of which 92 pathways were significantly enriched (*p* < 0.05) These included cell adhesion molecules (CAMs) (PATH:04514), insulin secretion (PATH:04911), cGMP-PKG signaling pathway (PATH:04022), metabolic pathways (PATH:01100), and calcium signaling pathway (PATH:04020). The top 45 significant GO terms and top 15 KEGG pathways are listed in Figure 5A,B, respectively, by ascending order of the corrected *p*-values.

### 3.5. The Comparative Analysis and Verification of the Expression Levels between Real-Time PCR Result and Sequencing Data

**Figure 6:**
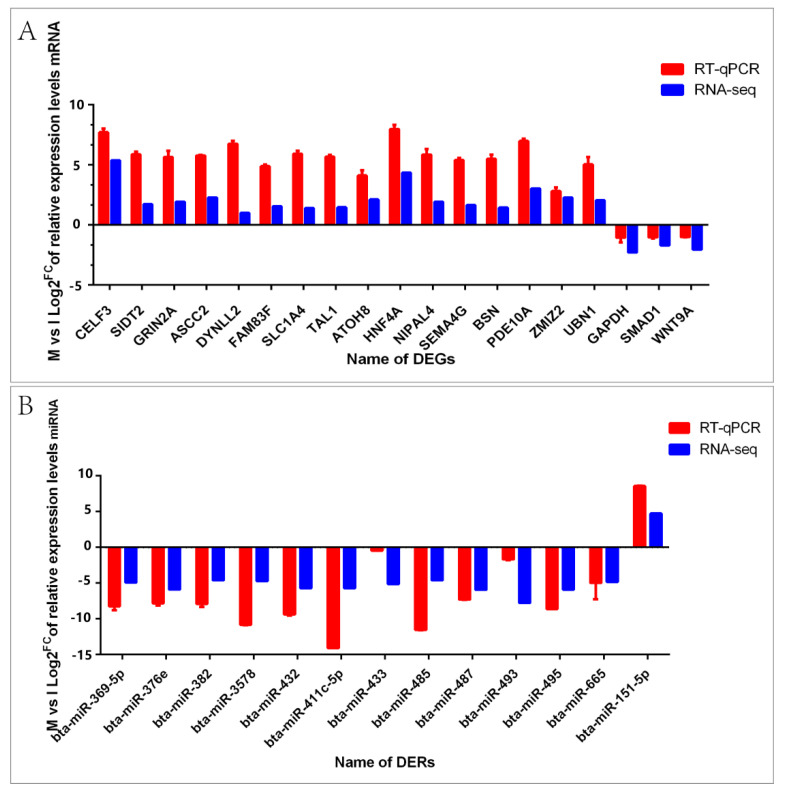
Fold change comparative analysis between real-time PCR result and sequencing data of DEGs and DERs. (**A**) The fold change of DEG expression levels; (**B**) The fold change of DER expression levels.

The partial DEGs and DERs were selected to validate the sequencing data by real-time PCR. Comparatively, the expression levels of activating signal co-integrator 1 complex subunit 2 (*ASCC2*), dynein light chain LC8-type 2 (*DYNLL2*), family with sequence similarity 83 member F (*FAM83F*), solute carrier family 1 member 4 (*SLC1A4*), TAL bHLH transcription factor 1 (*TAL1*), atonal bHLH transcription factor 8 (*ATOH8*), hepatocyte nuclear factor 4 alpha (*HNF4A*), NIPA like domain containing 4 (*NIPAL4*), semaphorin 4G (*SEMA4G*), bassoon presynaptic cytomatrix protein (*BSN*), phosphodiesterase 10A (*PDE10A*), zinc finger MIZ-type containing 2 (*ZMIZ2*), and ubinuclein 1 (*UBN1*) in mature testes were up-regulated, whereas glyceraldehyde-3-phosphate dehydrogenase (*GAPDH*), SMAD family member 1 (*SMAD1*) and Wnt family member 9A (*WNT9A*) were down-regulated (Figure 6A). In addition, bta-miR-369-5p, bta-miR-376e, bta-miR-382, bta-miR-3578, bta-miR-432, bta-miR-411c-5p, bta-miR-433, bta-miR-485, bta-miR-487, bta-miR-493, bta-miR-495 and bta-miR-665 had higher expression levels in mature testicular tissue. However, the expression level of bta-miR-151-5p was lower in mature testicular tissue (Figure 6B). The real-time PCR results were consistent with the sequencing data of RNA-seq and Solexa.

## 4. Discussion

### 4.1. Mapping and Length Distributions of the miRNAs Sequences

Bull breeding has considerable agricultural significance and semen quality has become an important research area for animal production and breeding topics including male fecundity trait, sperm preservation and artificial insemination of animals. Previous reports have indicated that dicer plays an important role in spermatogenesis [43,44]. MiRNAs cooperate with dicer to act as a posttranscriptional regulatory mechanism to participate in testicular tissue development and spermatogenesis [45,46]. Therefore, miRNAs are logical targets for exploring its effects on male fertility and may provide useful biomarkers for bull breeding. However, of the number of known bovine miRNAs only 1064 precursors and 1025 mature (Btau_5.0.1) were released in the miRBase database [39], which is currently much lower than that of humans (1917 precursors, 2654 mature (GRCh38)) and mice (1234 precursors, 1978 mature (GRCm38)). In the present study, some miRNAs were not defined in the bovine database. To further explore the functions of miRNAs, the novel miRNAs were mapped to the human and mouse databases and the results showed that some miRNAs in our bovine data had the same sequence with those found in humans and mice databases, such as hsa-miR-151b, hsa-miR-130a-5p, hsa-miR-3116, hsa-miR-4768-5p, mmu-miR-7035-5p, mmu-miR-351-5p, mmu-miR-7089-3p, and mmu-miR-3473d, indicating that bovine miRNAs may have homologous sequences with humans and mice, and a large number of bovine miRNAs need further study.

Mapping and length distribution of the miRNA sequences also found that the miRNAs of immature testicular tissue were predominantly distributed around 20nt to 24nt, which is typical of the small RNA of dicer-processed products. However, the miRNAs of mature testicular tissue mostly had a length of 22nt and 28nt in size. A study reported that the miRNAs with the length of 24-32nt typical piRNAs [47] predominantly expressed in sperm [48], indicating that our Solexa sequencing data included piRNAs of bovine sperm. Our previous study on porcine testes [49] is consistent with the data on the bull. The 20nt and 22nt sequences in the bovine testis of the 3d after birth and adult bull samples are the dominant small RNAs [49], while there are no 24-32nt piRNAs in the length distribution data of mature testicular tissue. Therefore, we speculated that the previous test just pools the same kinds of testicular tissue samples (the tissues of 3d after birth and adult bull) for Solexa sequencing, so the difference of length distributions between miRNAs and piRNA were not shown or neglected in the data.

### 4.2. Gene Expression Patterns and Roles in Testicular Tissues of Cattle

In the above, GO analysis of DEGs showed the down-regulated genes were significantly enriched in a large number of GO terms, and the GO tree also showed most GO terms were down-regulated, indicating that these DEGs may play important roles in the development of the immature testis of calves. However, the up-regulated genes enriched in GO terms were related to fertilization, male meiosis, meiotic, and protein ubiquitination of adult bulls, which indicate that a large number of genes with key roles in male reproduction traits are highly expressed in mature testis tissue, and these genes are not, or are lowly, expressed in immature testis tissue.

KEGG analysis showed a total of 7171 DEGs enriched in KEGG pathways, in which only 1109 genes were screened as up-regulated genes. A large number of genes with high expression in immature testis associated with growth and metabolism were more active, while high expression of genes in the mature testis were mainly located in the calcium signal metabolism pathway, cell cycle, and insulin signal pathway, which were related to cell proliferation, hormone regulation, and sperm development. The KEGG enrichment of DEGs indicated that a larger number of up-regulated genes were not defined in KEGG pathways which suggest that their functions in bull reproduction need to be explored in the future. Meanwhile, previous studies have shown that Sertoli proliferation and differentiation can be mediated through Wnt/β-catenin signaling pathway [50,51], mTOR signaling pathway [51,52,53,54], and TGF-β signaling pathway [55,56]. PTEN, PI3K/AKT, and STAT signaling pathways were found to be involved in bull sperm cell apoptosis [57]. In the present study, the above mentioned KEGG pathways, which have important effects on Sertoli proliferation and differentiation and bull sperm cell apoptosis, were enriched. Additionally, most DEGs enriched in metabolic pathways were involved in fat metabolism, and KEGG pathway enrichment analysis showed that pathways related to fat metabolism including fatty acid degradation (PATH:00071), adipocytokine signaling pathway (PATH:04920), and PPAR signaling pathway (PATH:03320) were significantly enriched. Several studies have reported that obesity decreases the fecundity of male and female reproduction in humans [58,59,60], which indicates that fat metabolism participates in reproductive regulation, which is consistent with our results. The present study suggested that fat metabolism also plays an important role in bovine reproduction. Although fat metabolism has always been the focus and hotspot of beef traits and milk quality traits research, recently studies of functional genes related to fat metabolism in reproduction have predominantly focused on female cattle [61,62,63,64,65]. Thus, the functions of DEGs enriched in fat metabolism pathways including peroxisome proliferator activated receptor alpha (*PPARA*), glycerol kinase (GK), acyl-CoA oxidase 2 (*ACOX2*), carnitine palmitoyl-transferase 1B (*CPT1B*), acyl-CoA synthetase bubblegum family member 2 (*ACSBG2*), etc., on male fertility should also be explored in future study. In addition, there were only 24 up-regulated pathways in networks, and it is noteworthy that both up- and down-regulated DEGs were significantly enriched in calcium signaling pathway and metabolic pathways. KEGG enrichment and pathway network suggested that the above two pathways play a vital and complex role in the development of testis and male trait maintenance. In general, the GO terms and the KEGG pathways for DEGs between mature and immature testicular tissue provide perfect clues for selecting candidates affecting bovine male reproduction.

Our study not only analyzed the networks with a large number of important pathways affect bovine male reproductions but also many other DEGs have been reported with functions in male fecundity or candidate genes in humans and mice. These DEGs include phosphoserine aminotransferase 1 (*PSAT1*), ubiquitin D (*UBD*), leucine rich repeat containing 32 (*LRRC32*), tsukushi, small leucine rich proteoglycan (*TSKU*), interferon-gamma (*IFN-γ*), and transcriptional activator GLI3-like (*GLI3*) [66,67]. One of the DEGs identified in the present study, podoplanin (*PDPN*), promotes the proliferation of immature bovine Sertoli cells in vitro in the research we have reported [68]. Other DEGs in the present analysis, such as coiled-coil domain containing 185 (*CCDC185*), protamine 2 (*PRM2*), serine protease 58 (*PRSS58*), BPI fold containing family A member 3 (*BPIFA3*), *SPACA4*, etc., were also reported as candidate genes for the regulation of male reproduction in cattle [69].

### 4.3. Target Predictions of DERs and Target Network

Many studies have indicated that miRNAs are important for the proliferation and/or early differentiation of stem cell populations in spermatogenesis [70]. Findings in previous studies have indicated the deletion of both miR-34b/c and miR-449a/b/c might lead to mice sterility. miR-34b/c reduced sperm count, changing sperm morphology, and abnormal motility [71], whereas miR-449a/b/c was essential for normal spermatogenesis and male fertility [72]. Meanwhile, miR-449a, miR-34c-5p, and miR-122 could be used as biomarkers of germ cell maturation [73,74]. Mouse miR-146 has also been shown to be associated with the differentiation state because its expression levels are markedly reduced in differentiating spermatogonia compared with undifferentiated cells [75]. The above mentioned miRNAs which have important functions in male reproduction in humans and mice were also identified in the present study, which indicated that these miRNAs play a vital role in bovine male reproduction.

In the present study, most miRNAs such as miR-34b, miR-34c, miR-449a, miR-449b, and miR-122 were closely related to cell proliferation, tissue development, and spermato-genesis. However, GO enrichment of miRNA target DEGs showed only 68 DEGs in male fertility related to spermatogenesis, sperm motility, spermatid development, sperm chromatin condensation, and positive regulation of sperm motility, which indicated that other known genes related to male reproduction did not currently depend on miRNA regulation to affect male reproduction. There were also 25 miRNAs target genes in GO terms related to male development and spermatogenesis, of which PTEN, PI3K/AKT, and STAT signaling pathways were found to be involved in bull sperm cells apoptosis and this process was affected by miR-122 dysregulation [57]. In our previous study, the expression of pyruvate dehydrogenase kinase 4 (*PDK4*)/dual specificity phosphatase 4 (*DUSP4*) and FKBP prolyl isomerase 1B (*FKBP1B*) could be regulated by miR-122 and miR-449, respectively, which play a vital role in regulating the proliferation and cell cycle in bovine Sertoli cells [76]. Nevertheless, there are still some miRNAs that are barely understood whose functions need to be explored in relation to male reproduction in the future.

Although our study chose three testicular lobules for each group to screen the regulators in Sertoli cells on spermatogenesis, we failed to isolate and sequence all specific functional regions of testicular tissues such as caput epididymis, corpus epididymis and cauda epididymis. Therefore, the differential genes and miRNAs obtained cannot fully summarize the expression patterns between mature and immature testicular tissues. Meanwhile, the candidate miRNAs and genes cannot obtain more specific classifications of testicular development and spermatogenesis functions. These issues will also need to be solved urgently in our future research.

## 5. Conclusions

Considering that miRNAs are also present in spermatozoa and seminal fluid, their stability and the relatively non-invasive procedures required to obtain these samples make miRNAs excellent candidates for use as biomarkers of male reproduction and fertility. This genome-wide integrated analysis of mRNAs and miRNAs in Chinese Red Steppes will aid in the ability to identify cattle fecundity performance among the bull population in the future. Our findings could also help to elucidate the weight of epigenetic regulation and to design excellent marker-assisted selection programs in cattle breeding, and identifying fertile males would be of financial benefit to the animal production industry.

## Figures and Tables

**Table 1:** The 15 most differentially expressed up- and 15 most differentially expressed down-regulated genes between the immature and mature testicular tissues.

AccID	Mature	Immature	Log2FC	FDR	Regulate
TRIM42	1754.37	0.00	20	0.00	up
SPACA4	879.07	0.00	20	0.00	up
FAM187B	776.40	0.00	20	0.00	up
LOC781895	693.90	0.00	20	0.00	up
PRPS1L1	634.28	0.00	20	0.00	up
LOC100335845	557.42	0.00	20	0.00	up
TMIGD3	423.84	0.00	20	0.00	up
LOC784318	405.71	0.00	20	0.00	up
CCDC182	396.56	0.00	20	0.00	up
LOC101904646	362.72	0.00	20	0.00	up
TMEM239	352.87	0.00	20	0.00	up
LOC101907601	339.97	0.00	20	0.00	up
LOC615451	280.92	0.00	20	0.00	up
LOC516636	232.22	0.00	20	0.00	up
LOC101906055	216.52	0.00	20	0.00	up
LOC104976276	0.00	68.67	−20	0.00	down
LOR	0.00	37.29	−20	0.00	down
LOC781553	0.00	34.92	−20	0.00	down
PDIA2	0.00	105.11	−20	0.00	down
CHRNA1	0.00	64.93	−20	0.00	down
LOC100847574	0.00	51.13	−20	0.00	down
MMP12	0.00	29.71	−20	0.00	down
LOC107133052	0.00	28.63	−20	0.00	down
MMP7	0.00	22.75	−20	0.00	down
LOC101904303	0.00	22.03	−20	0.00	down
LOC104970370	0.00	21.73	−20	0.00	down
GABRG3	0.00	16.98	−20	0.00	down
LOC104970773	0.00	16.71	−20	0.00	down
CSMD3	0.00	16.21	−20	0.00	down
LOC100139712	0.00	15.90	−20	0.00	down

**Table 2:** Total of 31 differentially expressed miRNAs in the immature and mature testicular tissue.

AccID	Mature	Immature	Log2FC	FDR	Style
bta-miR-449a	10,427.15	14.20	9.52	0.00	up
bta-miR-449b	326.43	1.49	7.77	0.00	up
bta-miR-34b	4001.49	35.13	6.83	0.00	up
bta-miR-34c	75,119.87	561.35	7.06	0.00	up
bta-miR-449c	151.18	1.49	6.66	0.00	up
bta-miR-146a	379.95	8.22	5.53	0.00	up
bta-miR-375	671.60	20.18	5.06	0.00	up
bta-miR-544b	28.09	0.00	20.00	0.01	up
bta-miR-2384	64.22	2.24	4.84	0.02	up
bta-miR-151-5p	13,120.22	488.10	4.75	0.02	up
bta-miR-2284s	17.39	0.00	20.00	0.04	up
bta-miR-493	1.34	290.77	−7.76	0.00	down
bta-miR-409b	2.68	224.24	−6.39	0.01	down
bta-miR-409a	0.00	94.93	−20.00	0.01	down
bta-miR-495	16.05	964.99	−5.91	0.01	down
bta-miR-487b	5.35	327.39	−5.94	0.01	down
bta-miR-376e	4.01	238.44	−5.89	0.01	down
bta-miR-411c-5p	6.69	346.08	−5.69	0.01	down
bta-miR-196b	0.00	69.51	−20.00	0.02	down
bta-miR-376b	1.34	92.69	−6.11	0.02	down
bta-miR-433	13.38	469.41	−5.13	0.03	down
bta-miR-3956	0.00	50.08	−20.00	0.03	down
bta-miR-432	255.53	13391.71	−5.71	0.03	down
bta-miR-541	0.00	44.85	−20.00	0.03	down
bta-miR-369-3p	9.36	284.79	−4.93	0.03	down
bta-miR-665	10.70	307.21	−4.84	0.04	down
bta-miR-496	0.00	38.87	−20.00	0.04	down
bta-miR-3578	16.05	417.84	−4.70	0.04	down
bta-miR-543	20.07	504.54	−4.65	0.05	down
bta-miR-382	17.39	419.33	−4.59	0.05	down
bta-miR-485	17.39	418.58	−4.59	0.05	down

## Data Availability

The datasets presented in this study can be found in online repositories. The names of the repository/repositories and accession number(s) can be found at: https://www.ncbi.nlm.nih.gov/geo/ (accessed on 31 December 2020), GSE137464.

## Supplementary Materials

The following are available online at https://www.mdpi.com/article/10.3390/ani11113024/s1, Table S1: The real-time PCR primers of DEGs and DERs, Table S2: Details of sequencing data by RNA-seq, Table S3: Details of sequencing data by Solexa.
