# Supplementary material for: Comprehensive Analysis of miRNAs and Target mRNAs between Immature and Mature Testis Tissue in Chinese Red Steppes Cattle
Source: Animals (Basel). 2021 Oct 21;11(11):3024. doi: 10.3390/ani11113024 (PMC8614260; doi:10.3390/ani11113024)
Supplement: Supplementary file 1 [file animals-11-03024-s001.zip › animals-1362968-supplementary.pdf]

Table S1. The real-time PCR primers of DEGs and DERs

Symbol	Sequence ID	Primer name	Sequence (5′-3′)
bta-miR-369-5p	MI0005020	RT-Primer	CTCAACTGGTGTCGTGGAGTCGGCAATTCAGTTGAGAAAGATCA
		F-Primer	ACACTCCAGCTGGGAATAATACATGGTTG
		R-Primer	TGGTGTCGTGGAGTCG
bta-miR-376e	MI0022290	RT-Primer	CTCAACTGGTGTCGTGGAGTCGGCAATTCAGTTGAGAATGTGGA
		F-Primer	ACACTCCAGCTGGGAACATAGAGGAAAATC
		R-Primer	TGGTGTCGTGGAGTCG
bta-miR-382	MI0009822	RT-Primer	CTCAACTGGTGTCGTGGAGTCGGCAATTCAGTTGAG CGAATCCA
		F-Primer	ACACTCCAGCTGGGGAAGTTGTTCGTGGTG
		R-Primer	TGGTGTCGTGGAGTCG
bta-miR-3578	MI0015945	RT-Primer	CTCAACTGGTGTCGTGGAGTCGGCAATTCAGTTGAG GAAGTTGT
		F-Primer	ACACTCCAGCTGGGGAATCCACCACGAAC
		R-Primer	TGGTGTCGTGGAGTCG
bta-miR-432	MI0009831	RT-Primer	CTCAACTGGTGTCGTGGAGTCGGCAATTCAGTTGAG CCACCCTT
		F-Primer	ACACTCCAGCTGGGTCTTGGAGTAGGTCATT
		R-Primer	TGGTGTCGTGGAGTCG
bta-miR-411c-5p	MI0022292	RT-Primer	CTCAACTGGTGTCGTGGAGTCGGCAATTCAGTTGAGAATGTATG
		F-Primer	ACACTCCAGCTGGGGGTTGATCAGAGAACA
		R-Primer	TGGTGTCGTGGAGTCG
bta-miR-433	MI0009832	RT-Primer	CTCAACTGGTGTCGTGGAGTCGGCAATTCAGTTGAG ACACCGAG
		F-Primer	ACACTCCAGCTGGGATCATGATGGGCTCCT
		R-Primer	TGGTGTCGTGGAGTCG

bta-miR-485	MI0009842	RT-Primer<br>F-Primer<br>R-Primer	CTCAACTGGTGTCGTGGAGTCGGCAATTCAGTTGAG CGAATTCA<br>ACACTCCAGCTGGGAGAGGCTGGCCGTGATG<br>TGGTGTCGTGGAGTCG
bta-miR-487b	MI0005060	RT-Primer<br>F-Primer<br>R-Primer	CTCAACTGGTGTCGTGGAGTCGGCAATTCAGTTGAGAAGTGGAT<br>ACACTCCAGCTGGGAATCGTACAGGGTCAT-3<br>TGGTGTCGTGGAGTCG
bta-miR-493	MI0009847	RT-Primer<br>F-Primer<br>R-Primer	CTCAACTGGTGTCGTGGAGTCGGCAATTCAGTTGAGCCTGGCAC<br>ACACTCCAGCTGGGTGAAGGTCTACTGTGT<br>TGGTGTCGTGGAGTCG
bta-miR-495	MI0009849	RT-Primer<br>F-Primer<br>R-Primer	CTCAACTGGTGTCGTGGAGTCGGCAATTCAGTTGAGAAGAAGTG<br>ACACTCCAGCTGGGAAACAAACATGGTGCA<br>TGGTGTCGTGGAGTCG
bta-miR-665	MI0009882	RT-Primer<br>F-Primer<br>R-Primer	CTCAACTGGTGTCGTGGAGTCGGCAATTCAGTTGAG AGGGGCCT<br>ACACTCCAGCTGGGACCAGTAGGCCGAG<br>TGGTGTCGTGGAGTCG
bta-miR-151-5p	MI0004738	RT-Primer<br>F-Primer<br>R-Primer	CTCAACTGGTGTCGTGGAGTCGGCAATTCAGTTGAGACTAGACT<br>ACACTCCAGCTGGGTCGAGGAGCTCACAG<br>TGGTGTCGTGGAGTCG
U6	NR_004394.1	RT-Primer<br>F-Primer<br>R-Primer	AACGCTTCACGAATTTGCGT<br>CTCGCTTCGGCAGCACA<br>AACGCTTCACGAATTTGCGT
CELF3	NM_001076845.1	F-Primer<br>R -Primer	CAACAGCAGCAGCAGCAA<br>ACATCTGGAGGATCTCTGAGTC

SIDT2	NM_001024530.3	F-Primer<br>R-Primer	GCTCTGTGGAGGAAGATGACTA<br>AGGTATTGCTTGGTGCGAATG
GRIN2A	XM_024985455.1	F-Primer<br>R-Primer	GCCTTCTTCGCCGTCATCT<br>GGAATAGTCGTGAGGTCTCTGAA
ASCC2	NM_001015524.1	F-Primer<br>R -Primer	TGAGTACGACGACACATACGA<br>TAGCCTCCTCCTCCTCTTCC
DYNLL2	NM_001113303.1	F-Primer<br>R-Primer	CACGCAGGCTATGGAGAAGTA<br>TCTTGATGTAGGCAGCAATGTC
FAM83F	XM_002687938.6	F-Primer<br>R-Primer	AACCTCCTCCTCCTCCTGAC<br>GCTGGTACAAGTTCACCTCCT
SLC1A4	NM_001081577.1	F-Primer<br>R-Primer	CTGGCTGTGGACTGGATTGT<br>TGATTCAGGTGGTGGAGGATG
TAL1	NM_001192495.2	F-Primer<br>R-Primer	GCGGCAGCAGAATGTGAAC<br>CCTTCCTCCTCCTGGTCATTG
ATOH8	NM_001193104.3	F-Primer<br>R-Primer	CAGGTGCCGTGTTACTCATAC<br>GCCAGGGACAGGATGTAGTT
HNF4A	NM_001015557.1	F-Primer<br>R -Primer	CCTCAGCAACGGACAGATGT<br>CTTGACGATGGTGGCAATAGC
NIPAL4	XM_015472345.2	F-Primer<br>R-Primer	ATCAGCACTCAGGTCAACTTC<br>TGGAGGAGGTCATCACTGTC
SEMA4G	NM_001101117.2	F-Primer<br>R-Primer	GTGACCAGCCATCCAACCT<br>TTCAGCATAGCAGCCGTAGT
BSN	XM_024983183.1	F-Primer<br>R-Primer	ACAGCGACAGCGACTACAG<br>CCAGGTCTGACTCCACATCC
PDE10A	XM_024997131.1	F-Primer<br>R-Primer	TTGATGATGACCGCCTGTGA

			CCTCGTCCTTCTTGTCTCTGT
ZMIZ2	NM_001205533.1	F-Primer	GCCATCCACCGACTTCACT
		R-Primer	GCTCCTGACTCTGCTTCTCC
UBN1	NM_001205922.1	F-Primer	TGAGCAGCGTGACATCGT
		R -Primer	GAAGACAGCAGGTTCAAGGAT
GAPDH	NM_001034034.2	F-Primer	GTTTGTGATGGGCGTGAAC
		R-Primer	ATGGACCTGGGTCATGAGT
SMAD1	NM_001076223.2	F-Primer	CCATTGGAATGCTGCGAGTT
		R-Primer	AGGCTGTGCTGAGGATTGTAT
WNT9A	XM_024995391.1	F-Primer	GCGAGGCTTCAAGGAGACA
		R-Primer	TTGACGAACTTGCTGCTGTAC
β-actin	NM_173979.3	F-Primer	AGAGCAAGAGAGGCATCC
		R-Primer	TCGTTGTAGAAGGTGTGGT

Table S2. Details of sequencing data by RNA-seq

Samples	Total Reads	Clean Reads	UniqueMapped	ReadsFilter%	UniqueMappedRat%
I1	52,246,126	51,617,112	44,214,723	0.988	85.7
I2	54,526,368	53,850,456	46,284,731	0.988	86.0
I3	54,526,368	53,857,862	46,081,945	0.988	85.6
M1	54,259,542	53,610,616	46,624,308	0.988	87.0
M2	53,161,792	52,538,338	45,130,866	0.988	85.9
M3	52,817,552	52,281,870	45,131,041	0.989	86.3

Samples: samples name; I1, I2, I3: immature testicular tissues of Chinese Red Steppes; M1, M2, M3: mature testicular tissues of Chinese Red Steppes.

Table S3. Details of sequencing data by Solexa

Samples	Total Reads	Clean Reads	UniqueMapped	ReadsFilter%	UniqueMappedRat%

I	6,000,000	5,354,600	97.2	0.892	35.5
M	6,000,000	5,737,270	98.5	0.956	83.1

Note: UniqueMapped: Unique mapped reads of the genome; UniqueMappedRat%: Unique mapped ratio of the genome.
